# Single-dose administration of therapeutic divalent siRNA targeting MECP2 prevents lethality for one year in an MECP2 duplication mouse model

**DOI:** 10.21203/rs.3.rs-6465542/v1

**Published:** 2025-04-25

**Authors:** Vignesh N. Hariharan, Ashley Summers, Amy E. Clipperton-Allen, Jillian Caiazzi, Samuel R. Hildebrand, Daniel O’ Reilly, Qi Tang, Zachary Kennedy, Dimas Echeverria, Nicholas McHugh, David Cooper, Jacqueline Souza, Chantal Ferguson, Laurent Bogdanik, Monica Coenraads, Anastasia Khvorova

**Affiliations:** 1 RNA Therapeutic Institute, University of Massachusetts Chan Medical School; Worcester, United States of America.; 2 The Jackson Laboratory; Bar Harbor, United States of America.; 3 The Rett Syndrome Research Trust; Trumbull, United States of America.

## Abstract

MECP2 duplication syndrome (MDS) is a rare X-linked neurodevelopmental disorder caused by duplications of the dosage-sensitive methyl-CpG-binding protein 2 (MECP2) gene. Developing effective therapies for MDS is particularly challenging due to the variability in MECP2 expression among patients and the potential risk of inducing Rett syndrome through excessive pharmacological intervention. Reducing dosage to optimize silencing levels often compromises durability and necessitates increased dosing frequency. We present here a series of fully chemically modified small interfering RNAs (siRNAs) designed for both isoform-selective and total MECP2 silencing. Among these, we identify six lead siRNA candidates across two distinct chemical scaffolds, achieving targeted total MECP2 expression reductions ranging from 25% to 75%, sustained for at least four months following a single administration. The efficacy and safety of human ortholog silencing were evaluated using two mouse models with distinct levels of human MECP2 transgene expression. In the severe duplication model, a single dose of the total isoform-silencing siRNA fully rescued early mortality and behavioral impairments. Additionally, we show that the isoform-selective targeting strategy may be safer in mild cases of MDS where exaggerated pharmacology may lead to Rett Syndrome. Overall, this study introduces a series of preclinical candidates with the capacity to address the varying levels of MECP2 duplication encountered in clinical settings. Furthermore, it establishes a target selection strategy that may be applied to other dosage-sensitive gene imbalances.

## One Sentence Summary

Therapeutic siRNAs provide safe and durable modulation of MECP2 for the treatment of mild and severe MECP2 Duplication Syndrome.

## INTRODUCTION

Methyl-CpG-binding protein 2 (MECP2) duplication syndrome (MDS) is a rare X-linked neurodevelopmental disorder caused by duplication of Xq28 encompassing the dosage-sensitive MECP2 gene. Precise *MECP2* dosage is critical: its underexpression causes Rett syndrome (RTT), while overexpression leads to MDS^1 2^. Nucleic acid-based therapeutics (NATs), particularly antisense oligonucleotides (ASOs) and siRNAs, offer targeted gene silencing via RNA interference and hold promise for MDS. ION440, an ASO currently in clinical trials (NCT06430385), is the most advanced candidate^3,4^. The therapeutic challenges of using NATs center on their pharmacodynamics. Titration of the dose to avoid over-silencing may compromise the duration of effect^5–7^. Reduced dosage to avoid over-silencing necessitates more frequent intrathecal administration, increasing the risk of injection related adverse events^8–11^. The variability in MECP2 expression levels among patients further complicates dosing strategies^12^.

Alternative splicing of *MECP2* produces two isoforms, MECP2 E1 and MECP2 E2, which share identical DNA-binding and functional domains. MECP2 E1 comprises the majority of MECP2 in neurons and has a lower DNA-binding affinity compared to MECP2 E2^13,14^. MECP2 E1 ablation is sufficient to cause RTT like symptoms in mice while MECP2 E2 ablation is well tolerated in the context of RTT^15,16^. Additionally, MECP2 E1 mutations have been found in RTT patients while MECP2 E2 mutations have not been seen in the clinic^17–19^ suggesting that MEPC2 E1 loss-of-function is the main driver of the RTT phenotype. However, transgenic expression studies have demonstrated that either MECP2 E1 or MECP2 E2 can rescue the lethal phenotype and behavioral abnormalities observed in MECP2-null mice in a dose dependent, isoform independent manner^14,20–23^. A possible explanation for the apparent contradiction—that MECP2 E1 deletion causes RTT, yet transgenic MECP2 E2 rescues it—is that E1 is more abundantly expressed and translated, comprising ~90% of brain MECP2^24^. Thus, studies showing E1 deletion alone induces RTT may reflect isoform expression differences rather than true functional non-redundancy. In MDS, the specific contributions of E1 and E2 remain unstudied, leaving it unclear whether overexpression of either isoform alone is sufficient to cause disease. Therefore, in the context of MDS, selective maximal silencing of one isoform could reduce the overall total MECP2 expression level without dose titration.

An emerging class of NATs particularly well-suited to addressing the challenge of tunable yet durable silencing of MECP2 involves fully chemically modified siRNAs. We showed previously that divalent, fully chemically modified asymmetric siRNAs achieve broad central nervous system (CNS) distribution in mice and non-human primates (NHPs), enabling sustained gene silencing with a wide therapeutic index^5^. In this study, we developed six fully chemically modified divalent siRNA therapeutic candidates capable of tunably silencing total MECP2 or MECP2 E1. Using this panel of siRNAs, we show complete reversal of behavioral phenotypes and rescue of early mortality in a severe MDS mouse model as well as safe gene modulation without negative behavioral impact in a milder mouse model of MDS. The identified pre-clinical candidates are cross-reactive to seven potential preclinical species, paving the way for formal pre-clinical development.

## RESULTS

### In vitro screen identifies potent multi-species targeting lead siRNAs

A comprehensive bioinformatic analysis of MECP2 transcripts, including the 5’ and 3’ untranslated regions (UTRs) and the open reading frame (ORF), was conducted to identify potential active siRNA sequences, following established guidelines^25,26^. Twenty-four top-scoring sequences, including those human-mouse cross-reactive sites, were synthesized for in vitro screening. All siRNAs were synthesized using fully chemically modified, asymmetric siRNA scaffolds with 2’-fluoro, 2’-O-methyl, and phosphorothioate modifications, as previously described^27^. To facilitate gymnotic uptake into cells without transfection reagents, the 3’ terminus of the passenger strand was covalently linked to cholesterol. Details of all sequences and chemical modification patterns used in this study are provided in the supplementary material (Table S1).

**Figure. 1.**
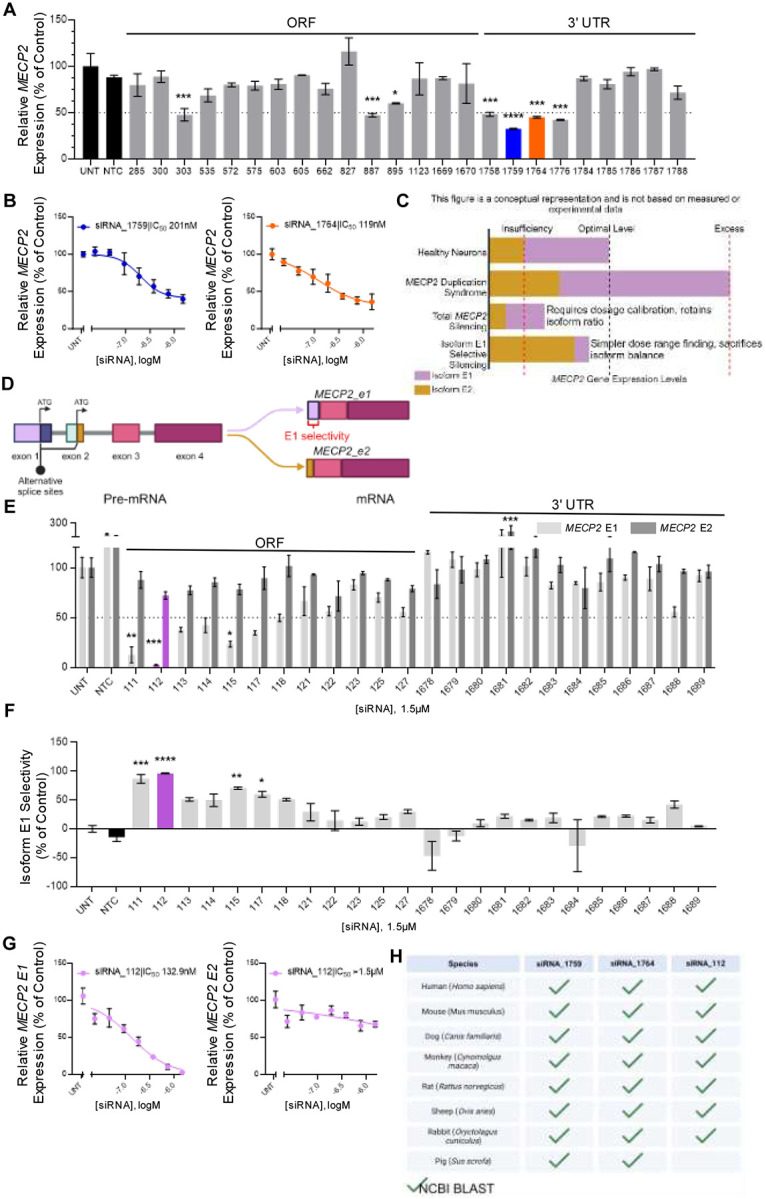
Lead total and isoform selective MECP2 silencing siRNAs identified through *in vitro* screens. (A) Relative expression of total human *MECP2* mRNA in HeLa cells 72h after treatment with non-isoform selective *MECP2* targeting siRNA at a single concentration. Lead compounds selected for further analysis are shown as blue (1759) and orange (1764) bars. (B) Relative expression of total human *MECP2* mRNA in HeLa cells 72h after treatment with total isoform silencing lead siRNAs 1759 (blue) and 1764 (orange) across a range of concentrations. (C) Potential strategies for modulation of MECP2 without dosage related toxicity. (D) Schematic of alternative splicing of the MECP2 pre-mRNA. (E) Relative expression of *MECP2-E1* (light grey bars) and *MECP2-E2* (dark grey bars) mRNA in HeLa cells 72h after treatment with E1 isoform selective siRNA at a single concentration. Lead compound (112) selected for further analysis is shown in purple bars. (F) Isoform E1 selectivity of siRNA compounds screened in (E). (G) Relative expression of *MECP2-E1* mRNA in HeLa cells 72h after treatment with E1 isoform selective siRNA 112 across a range of concentrations. (H) Results of cross-reactivity analysis of the targeting regions of lead siRNAs across the genome of humans and pre-clinical species using Basic Local Alignment Search Tool (BLAST) from the National Center for Biotechnology Information (NCBI) (green checkmarks) database. Gene expression was measured using the QuantiGene^™^ Singleplex Assay Kit. Data represented as mean±s.e.m. of three independent replicates. Statistical analysis performed using Ordinary one-way ANOVA with Dunnett’s adjustment for multiple comparisons (*-p<0.05, **-p<0.01, ***-p<0.001, ****-p<0.0001).

siRNA candidates targeting all MECP2 mRNA isoforms were screened in vitro at a single concentration of 1.5μM in human HeLa cells (Figure 1A) and mouse N2A cells (Supplemental Figure S1A), with gene expression assessed using the Quantigene 2.0 branched DNA assay. A non-targeting control siRNA (NTC) served as a negative control in all in vitro screens. siRNAs 1759 and 1764 exhibited significant *MECP2* silencing and were selected for further potency evaluation in both HeLa cells (Figure 1B) and N2A cells (Supplemental Figure S1B). The IC_50_ values for two leads identified, siRNA_1759 and siRNA_1764 were 201nM and 119nM, respectively, in passive uptake in HeLa cells, with comparable potency observed in N2A cells. These results indicate that these siRNAs are suitable candidates for further *in vivo* evaluation.

Given that MECP2 is a dosage-sensitive gene, we explored a strategy to achieve tunable silencing without sacrificing therapeutic durability (Figure 1C). The two major MECP2 isoforms, E1 and E2, may have sufficient functional overlap to provide redundancy^20^. In MDS, duplications typically span multiple genes and both isoforms are overexpressed, resulting in elevated total MECP2 levels. We hypothesize that silencing E1 to maximal achievable levels, without compromising dosage or duration, could reduce total MECP2 levels to within the optimal range. Isoform E2, which would remain over-expressed, should preserve sufficient MECP2 function.

We designed a panel of human and human-mouse cross-reactive siRNAs targeting exon 1, which is present in isoform E1 but absent in E2 (Figure 1D). HeLa cells were treated with 1.5μM of cholesterol-conjugated siRNAs designed to target MECP2 E1 without affecting E2. Expression levels of isoforms E1 and E2 were then measured using custom isoform-specific probes with the Quantigene 2.0 branched DNA assay. siRNA_112 provided maximal silencing of E1 (Figure 1E), with a ~95% selectivity for E1 over E2 (Figure 1F). We were unable to find any siRNAs that specifically silenced the E2 isoform without affecting E1 (Supplementary Figure S1C). This result was corroborated in seven-point dose-response assays, where siRNA_112 silenced MECP2 E1 mRNA with an IC_50_ of 133nM in HeLa cells (Figure 1G) and 263nM in N2A cells (Supplemental Figure S1D), with minimal impact on MECP2 E2 mRNA expression.

The targets of the lead siRNAs—1759, 1764, and 112—were completely conserved across seven pre-clinical species (Figure 1H). The cross-species homology of these lead compounds ensures that formal IND-enabling preclinical studies will not be limited by the availability of on-target pharmacology models.

### Lead MECP2-targeting siRNAs tune MECP2 levels in wildtype mice for up to four months

**Figure. 2.**
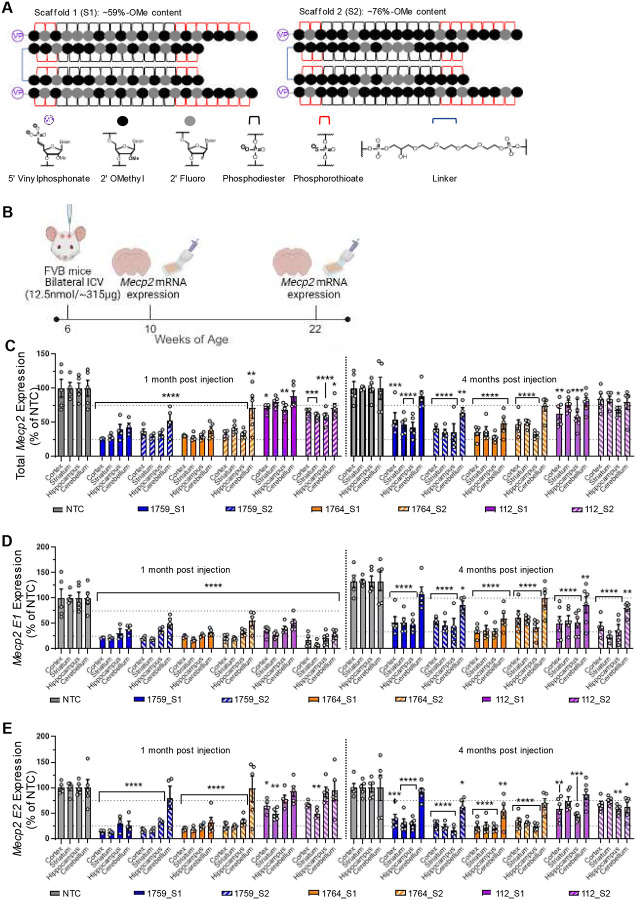
Lead compounds show potent and sustained modulation of *Mecp2* mRNA expression in the wildtype mouse brain. (A) Schematic of divalent scaffolds, modification patterns and chemical structures of relevant modifications used in this experiment. (B) Illustration of study design including dosage, route of administration, timeline and readouts. (C) Total *Mecp2* mRNA expression levels, (D) *Mecp2-E1* mRNA expression levels and (E) *Mecp2-E2* mRNA expression levels in the cortex, striatum, hippocampus and cerebellum of wildtype FVB mice injected with 12.5nmol (~315 μg) of siRNA variants. Gene expression was measured at one month and four months post injection using the QuantiGene^™^ Singleplex Assay Kit. Data represented as mean±s.e.m. of individual animals (n=4–6 mice/group). Statistical analysis performed using two-way ANOVA with Dunnett’s adjustment for multiple comparisons (*-p<0.05, **-p<0.01, ***-p<0.001, ****-p<0.0001).

To evaluate the in vivo potency and duration of effect for lead siRNAs, we synthesized these siRNAs using a divalent brain delivery scaffold to enable deep brain distribution and multi-month silencing across species^5^. The divalent siRNAs, composed of two siRNA passenger strands linked by a tetraethylene glycol linker and hybridized to the guide strand, were synthesized in two distinct modification patterns: scaffold 1 (~59% 2’-O-methyl content) and scaffold 2 (~76% 2’-O-methyl content), both of which have been previously demonstrated to modulate silencing for other siRNA sequences^28–30^. The siRNAs also incorporated gold standard 5’vinylphosphonate (VP) and phosphorothioate (PS) ends for added stability^31–33^ (Figure 2A).

Female wildtype FVB mice were administered 12.5 nmol (~315 μg) of divalent siRNAs or divalent non-targeting control (NTC) siRNA via intracerebroventricular (ICV) injection at six weeks of age. Mice were sacrificed at one- and four-months post-injection, and total Mecp2, Mecp2-E1, and Mecp2-E2 mRNA expression levels were analyzed in the cortex, striatum, hippocampus, and cerebellum using the Quantigene 2.0 branched DNA assay (Figure 2B). E1 and E2 levels were employed to assess siRNA selectivity, while the total Mecp2 levels served as the primary metric of pharmacodynamics.

At one-month post-injection, total Mecp2 mRNA levels in the cortex, striatum, and hippocampus of mice treated with non-isoform-selective siRNAs (1759_S1, 1759_S2, 1764_S1, and 1764_S2) were reduced to ~25–35% of NTC controls (p<0.0001), with no significant differences observed across these brain regions (Figure 2C). Silencing in the cerebellum was less pronounced and more variable, due to lower siRNA accumulation in this region of rodent brains (6). In the cerebellum, mice treated with 1759_S1 and 1764_S1 showed mean total Mecp2 expression levels of 43% (p<0.0001) and 38% (p<0.0001), respectively. In contrast, the 2’-O-methyl-rich scaffold 2 siRNAs (1759_S2 and 1764_S2) resulted in higher Mecp2 levels of 53% (p<0.0001) and 71% (p<0.01), respectively. At four months post-injection, 1764_S1 performed best and retained near-maximal silencing in all brain regions tested, with total Mecp2 mRNA levels between 35%−48% (p<0.0001) across all brain regions tested. For isoform E1-selective siRNAs (112_S1 and 112_S2), total Mecp2 mRNA levels were reduced to 59%−72% across brain regions with this effect being maintained for at least four months. While 112_S2 showed better performance at one month, 112_S1 demonstrated superior durability for the duration tested (Figure 2C).

At one month post injection, differences in E1 silencing between scaffolds S1 and S2 for 1759 and 1764 were more pronounced in the cerebellum, confirming that scaffold S2 was less active than S1 for 1759 and 1764 siRNAs, while 112_S2 remained the most effective at silencing E1 mRNA in the cerebellum. At four months post-injection, E1 mRNA levels across brain regions and compounds were 17%–44%. Although 112_S2 showed greater silencing at one month, 1764_S1 exhibited slightly, though not statistically significant, better durability in the cortex and hippocampus. This difference was more evident in the cerebellum, indicating that 1764_S1 provided the most durable silencing of Mecp2-E1 (Figure 2D).

At one-month post-injection, suppression of Mecp2 isoform E2 mRNA was notably higher than that of E1 or total Mecp2 for the non-isoform-selective siRNAs (<25% of NTC). E1-selective siRNAs 112_S1 and 112_S2 also reduced E2 expression levels in the cortex and striatum but not the hippocampus and cerebellum, indicating that these siRNAs did not achieve full E1 isoform selectivity at the tested doses in certain brain regions. Scaffold S2 demonstrated less silencing that S1 in the cerebellum. At four months post-injection, 112_S1 and 112_S2 had some impact on E2 mRNA levels, but this effect did not reach statistical significance except in the hippocampus. The effect of non-selective 1759 and 1764 were similar to that observed at the one month time point, with no significant silencing observed in mice treated with 1759_S1, 112_S1, or 1764_S2 (Figure 2E).

**Figure. 3.**
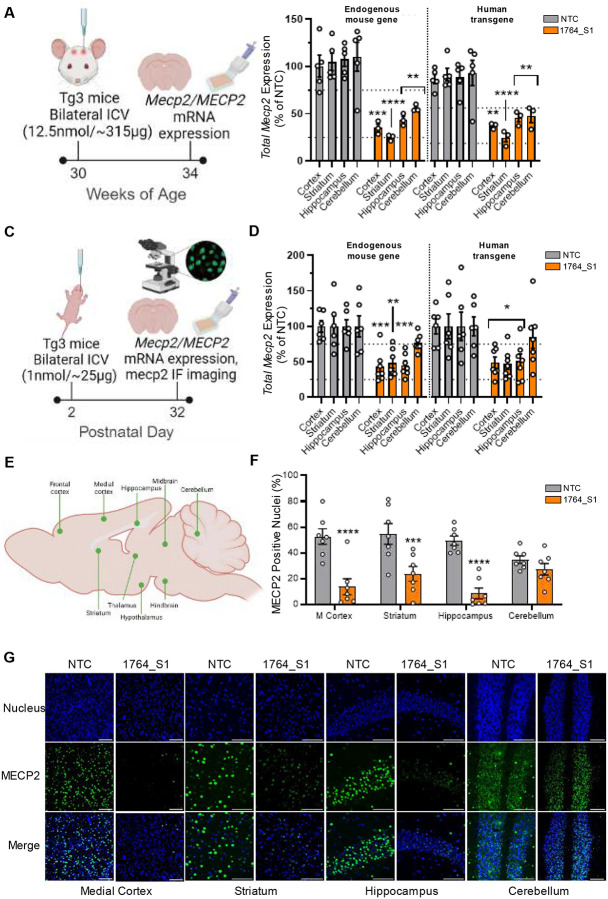
1764_S1 is active against mouse and human MECP2 in the adult and neonatal Tg3 brain. (A) Illustration of study design including dosage, route of administration, timeline and readouts in adult Tg3 mice. (B) Total mouse *Mecp2* and human mouse *MECP2* mRNA expression levels in the cortex, striatum, hippocampus and cerebellum of adult Tg3 mice injected with 12.5nmol (~315 μg) of 1764_S1 (n=5 mice for NTC and n=3 mice for 1764_S1). (C) Illustration of study design including dosage, route of administration, timeline and readouts in neonatal Tg3 mice. (D) Total mouse *Mecp2* and human mouse *MECP2* mRNA expression levels in the cortex, striatum, hippocampus and cerebellum of neonatal Tg3 mice injected with 1nmol (~25 μg) of 1764_S1 (n=6 for NTC and n=7 for 1764_S1). (E) Illustration of brain regions of interest identified for relative quantification of immunofluorescence from mice shown in (C). (F) Percentage of MECP2 positive nuclei in the medial cortex (M. cortex), striatum, hippocampus and cerebellum of neonatal mice shown in (C). (G) Representative immunofluorescence images at 40x magnification of samples quantified in (F), scale bar = 100μm. Gene expression was measured using the QuantiGene^™^ Singleplex Assay Kit. Data represented as mean±s.e.m. of individual animals. Immunofluorescence imaging was performed on sagittal brain sections stained with DAPI and anti-MECP2 antibody and imaged using Leica DMi8 widefield microscope. Quantification of MECP2 positive nuclei was performed using a custom imageJ script. Statistical analysis performed using two-way ANOVA with Dunnett’s adjustment for multiple comparisons (*-p<0.05, **-p<0.01, ***-p<0.001, ****-p<0.0001).

In general, all non-selective compounds maintain robust silencing of total Mecp2 with less than 30–40% of total mRNA remaining at up to four-months post injection. There were no significant differences between scaffolds except for 1764_S1 showing higher potency in the cerebellum compared to 1764_S2. Based on this data, 1764 S1 was selected for further evaluation in disease models. The isoform selective compound 112_S1 was selected as a second lead compound to evaluate the effects of E1 silencing with minimal perturbation of E2 levels. We have previously seen differences between protein and mRNA silencing for CNS targets due to the presence of inaccessible nuclear pools of target mRNA^34^. Indeed, we later evaluated the MECP2 silencing in the context of the animal models and confirmed that ~ 60–70% mRNA silencing corresponds to highly potent protein reduction of ~ 80–90% (Figure 3). Notably, reducing MECP2 protein by 70–80% in wild-type brains produced no overt toxicity, though this study was not designed to detect subtle behavioral changes. Prior work shows even 20–30% reductions can induce anxiety-like and RTT-like behaviors^35^, warranting further investigation.

### Activity of 1764_S1 against human MECP2 confirmed in a severe transgenic mouse model of MDS

To account for the variability in MECP2 levels in MDS, we selected two human transgenic MDS models developed by the Zoghbi lab^36^—MECP2Tg1 (Tg1), which expresses MECP2 at approximately two-fold the wild-type level, and MECP2Tg3 (Tg3), which expresses MECP2 at approximately three- to five-fold the wild-type level—to evaluate the efficacy of lead siRNAs. We verified the in vivo efficacy of 1764_S1 for silencing human MECP2 in the Tg3 transgenic line before proceeding to test both lead siRNAs for their potential to modulate MDS in Tg1 and Tg3 models.

Adult Tg3 mice at 30 weeks of age were injected with 12.5 nmol (~315 μg) of 1764_S1 via bilateral intracerebroventricular (ICV) injection. The mice were sacrificed at 34 weeks of age, and their brains were analyzed for total MECP2 mRNA levels (Figure 3A). We measured the total MECP2 mRNA of both species using species-specific probesets in the QuantiGene^™^ Singleplex branched DNA assay. Total mouse Mecp2 mRNA levels in the cortex, striatum, hippocampus, and cerebellum were 35% (p < 0.001), 25% (p < 0.0001), 44% (p < 0.01), and 56% (p < 0.01) of non-targeting control (NTC) levels, respectively. Silencing of total human MECP2 mRNA was slightly lower, with levels of 50% (p < 0.01), 33% (p < 0.0001), 61% (p < 0.01), and 64% (p < 0.01) in the cortex, striatum, hippocampus, and cerebellum, respectively (Figure 3B).

Since early intervention for MDS may be beneficial, we assessed whether silencing could be achieved in the smaller brains of neonatal mice. Tg3 mice at postnatal day 2 were injected with 1 nmol (~25 μg) of 1764_S1 via manual bilateral ICV injection, and the mice were sacrificed at postnatal day 32. Total human and mouse MECP2 mRNA and total MECP2 protein levels were analyzed (Figure 3C). Total mouse Mecp2 mRNA levels in the cortex, striatum, hippocampus, and cerebellum were 43% (p < 0.001), 48% (p < 0.01), 45% (p < 0.001), and 77% (p = 0.3) of NTC controls, respectively. Total human MECP2 mRNA levels in the cortex, striatum, hippocampus, and cerebellum were 49% (p < 0.01), 47% (p < 0.01), 51% (p < 0.01), and 85% (p = 0.9) of NTC controls, respectively (Figure 3D).

For immunofluorescence quantification, whole brain tiled scans were collected at 40x magnification. Brain regions were identified and cropped for analysis (Figure 3E). Image thresholding was used to accurately separate the DAPI (nucleus) fluorescence signal from the background, followed by quantification using the ImageJ Measure Function. Individual nuclei were identified and counted as regions of interest (ROIs), and within each ROI, MECP2-positive nuclei were counted and expressed as a percentage of the total nuclei in each brain region. On average, 500 to 1,000 nuclei were identified for each brain region per animal. MECP2 protein positivity was significantly lower in 1764_S1-treated mice compared to NTC controls across most regions measured. The percentage of MECP2-positive nuclei for each region, expressed as 1764_S1 vs. NTC, was as follows: 10% vs. 50% (p < 0.0001) in the prefrontal cortex, 24% vs. 55% (p < 0.001) in the striatum, 14% vs. 53% (p < 0.0001) in the medial cortex, 8% vs. 50% (p < 0.0001) in the hippocampus, 18% vs. 47% (p < 0.01) in the thalamus, 26% vs. 52% (p < 0.01) in the hypothalamus, 35% vs. 50% (p = 0.3) in the midbrain, 30% vs. 49% (p = 0.1) in the hindbrain, and 27% vs. 35% (p = 0.9) in the cerebellum (Figure 1F and Supplemental Figure S2F). Representative immunofluorescence images of each brain region from NTC and 1764_S1-treated mice are shown in Figure 3G and Supplemental Figures S2A–E.

Protein silencing was higher than mRNA silencing, potentially due to nuclear mRNA localization as reported for other targets^34^.

### Single injection of non-isoform selective 1764_S1 but not E1 selective 112_S1 induces mixed behavioral effects in Tg1 mice

To evaluate the efficacy of the lead compounds in modifying disease and the potential for Rett-like toxicity due to excessive suppression, we utilized the mild Tg1 model of MDS. This model is known to overexpress the human MECP2 transgene at approximately twice the levels observed in wild-type FVB mice. MECP2 overexpression was confirmed by JAX prior to the study, with Western blot data indicating an ~2.4-fold increase in MECP2 expression compared to FVB wild-type controls (Supplemental Figure S3A). Behavioral assays were chosen based on published data from MDS transgenic mouse models and were conducted using standard operating procedures established by JAX In Vivo Pharmacology Services. The study was performed by JAX.

**Figure 4.**
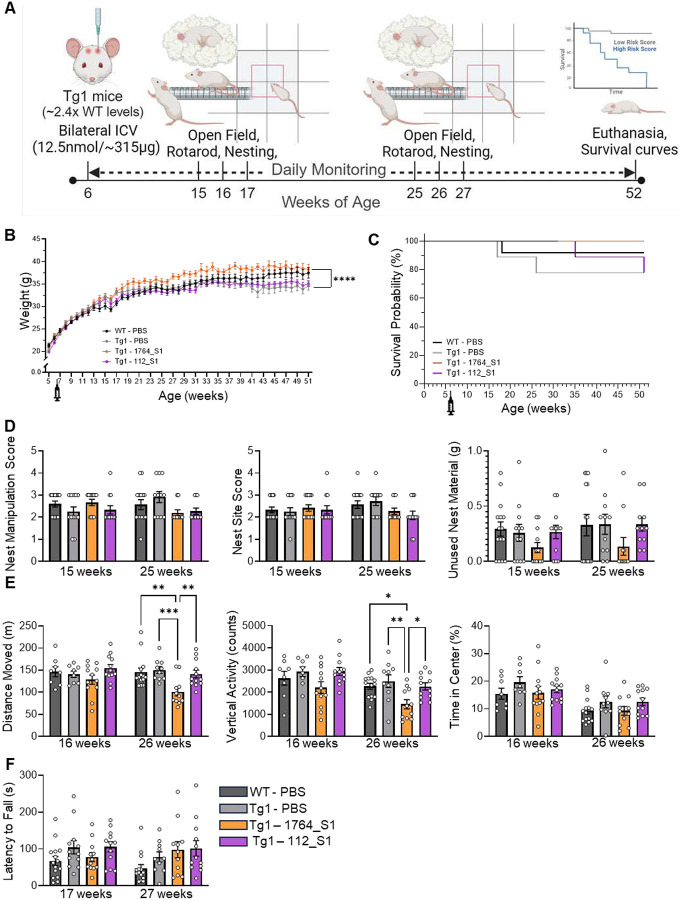
1764_S1 but not 112_S1 induces mixed behavioral effects in Tg1 mice. (A) Illustration of study design including dosage, route of administration, timeline and readouts in WT and Tg1 mice treated with PBS (black data points – WT, grey data points – Tg1), 1764_S1 (orange data points) and 112_S1 (purple data points). (B) Weight of treated animals over time. (C) Mortality of treated animals over time expressed as the probability of survival. (D) Nest building assay scores including nest manipulation score (left), nest site score (middle) and unused nesting material (right) measured at 15 and 25 weeks of age. (E) Open field test results including distance moved (left), vertical activity counts (middle) and time in the center (right) measured at 16 and 26 weeks of age. (F) Latency to fall on the rotarod test measured at 17 and 27 weeks of age. Data represented as mean±s.e.m. of individual animals (n=12–15 mice per group). Statistical analysis performed using two-way ANOVA with Dunnett’s adjustment for multiple comparisons (**-p<0.01, ****-p<0.0001).

Adult six-week-old male Tg1 mice were administered 12.5 nmol (~315 μg) of either 1764_S1, 112_S1, or phosphate-buffered saline (PBS) as a vehicle control via intracerebroventricular (ICV) injection. We elected to use a single PBS control group as we have previously established the equivalency of NTC and PBS on target silencing in vitro (see Fig. 1A, E and F) and in vivo^5^. Wild-type littermates received PBS as a negative control for the MDS genotype. Technicians were blinded to the content of each test article throughout the study. Post-administration, the mice were monitored for up to a year before euthanasia. Behavioral assessments included nest building at 15 and 25 weeks of age, the Open Field Test at 16 and 26 weeks of age, and rotarod performance at 17 and 27 weeks of age (Figure 4A). These timepoints were selected to evaluate behavior at a reasonable onset age and to track disease progression based on previous publications^36^. The experiment aimed to assess the potential of lead siRNAs to alter behavior and mortality in MDS mice; tissue samples were not collected for molecular biology analyses before euthanasia.

The body weight of Tg1 mice administered PBS was comparable to that of wild-type FVB (WT) mice given PBS, indicating no genotype effects on weight or weight gain in this experiment. Tg1 mice treated with the E1 isoform-selective 112_S1 also showed similar weights to Tg1 PBS and WT PBS mice. Tg1 mice treated with the non-isoform-selective 1764_S1 exhibited a statistically significant weight gain compared to Tg1 PBS controls, with the two groups diverging in weight after 16 weeks of age (Figure 4B). Survival analysis revealed no significant differences in survival up to 52 weeks of age for either WT or Tg1 mice, and no treatment effects were observed on mortality (Figure 4C). The reason for this discrepancy in mortality compared to previous reports^36^ is unclear as protein analysis of WT and Tg1 brains at four weeks of age confirmed transgene overexpression. One potential explanation is that in this study, mice were separated and individually housed due to excessive fighting which may have prevented aggression related mortality. Increased aggression in male mice with ~50% transgenic overexpression of Mecp2 in the FVB but not in the C57BL/6 background has been reported previously^37^.

Nest building, previously reported in Rett and MDS mice^37,38^, was assessed by providing each mouse with a single nestlet in their home cage. After 24 hours, nest photographs were scored based on nest manipulation and site quality, and the weight of unused nest material was recorded. At both 15 and 25 weeks of age, transgene overexpression did not significantly impact nest-building parameters and treatment with isoform selective 112_S1 did not significantly change nest building. Treatment with non-isoform selective 1764_S1 resulted in over-manipulation of nests compared to wildtype and vehicle treated Tg1 mice (Figure 4D). In the Open Field Test, conducted at 16 and 26 weeks of age, Tg1 PBS animals did not exhibit any change in behavior compared to wildtype controls. Treatment of Tg1 mice with isoform selective 112_S1 did not alter this behavior. Treatment with the non-isoform selective 1764_S1 significantly reduced the distance moved and vertical activity at 26 weeks of age (Figure 4E). The rotarod test, administered at 17 and 27 weeks of age, showed no differences in the latency to fall between any of the groups tested (Figure 4F).

Collectively, these results suggest that in our study, the Tg1 MDS mice did not experience severe disease, and showed no changes in survival probability or behavior relative to WT controls. However, treatment with the non-isoform-selective 1764_S1 did significantly increase body weight, decrease activity in the Open Field, and possibly increase manipulation of nest material in the nest building test. These behavioral differences in 1764_S1, but not 112_S1 treated Tg1 mice indicate that isoform selective silencing is better tolerated in this model.

### A single administration of siRNA modifies disease in severe MDS mice

Given the lack of a clear MDS phenotype in Tg1 mice, we turned to a more severe MDS model, the Tg3 mice, to evaluate the effectiveness of MECP2 silencing siRNA in disease modification. This model reportedly overexpresses the human MECP2 transgene at approximately 3.5-fold higher levels than wild-type FVB mice. MECP2 overexpression was confirmed by JAX before the study, with Western blot data showing an ~8.4-fold increase in MECP2 expression compared to FVB wild-type controls (Supplemental Figure S3B). Behavioral assays were conducted using standard operating procedures established by JAX In Vivo Pharmacology Services. The study was conducted at JAX.

**Figure 5.**
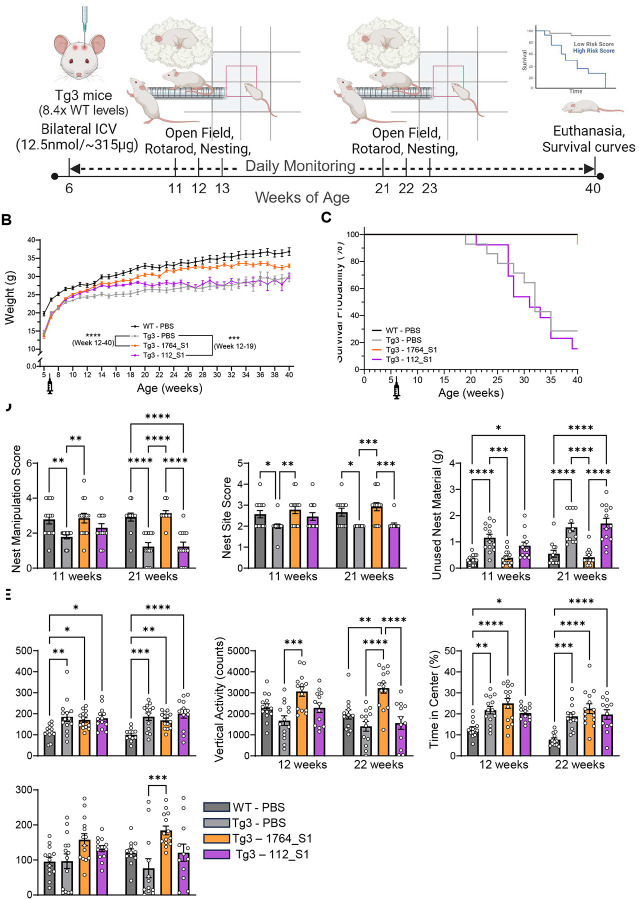
112_S1 partially and 1764_S1 completely rescues severe MDS in Tg3 mice. (A) Illustration of study design including dosage, route of administration, timeline and readouts in WT and Tg3 mice treated with PBS (black data points – WT, grey data points – Tg1), 1764_S1 (orange data points) and 112_S1 (purple data points). (B) Weight of treated animals over time. (C) Mortality of treated animals over time expressed as the probability of survival. (D) Nest building assay scores including nest manipulation score (left), nest site score (middle) and unused nesting material measured at 11 and 21 weeks of age. (E) Open field test results including distance moved (left), vertical activity counts (middle) and time in the center measured at 12 and 22 weeks of age. (F) Latency to fall on the rotarod test measured at 13 and 23 weeks of age. Data represented as mean±s.e.m. of individual animals (n=12–14 mice per group). Statistical analysis performed using two-way ANOVA with Dunnett’s adjustment for multiple comparisons (*-p<0.05, **-p<0.01, ***-p<0.001, ****-p<0.0001).

Adult six-week-old male Tg3 mice were administered 12.5 nmol (~315 μg) of either 1764_S1, 112_S1, or phosphate-buffered saline (PBS) as a vehicle control via intracerebroventricular (ICV) injection. Wild-type littermates received PBS as a negative control for the MDS genotype. Technicians were blinded to the contents of each test article throughout the study. The mice were monitored for up to 40 weeks post-administration before euthanasia. Behavioral assessments included nest building at 11 and 21 weeks of age, the Open Field Test at 12 and 22 weeks of age, and rotarod performance at 13 and 23 weeks of age (Figure 5A). These time points were chosen to evaluate behavior at a reasonable onset age and to track disease progression; tissue samples were not collected for molecular biology analyses before euthanasia.

Tg3 male mice started at lower body weights than WT males. Both the non-isoform-selective 1764_S1 and the E1 isoform-selective 112_S1 increased the body weight of Tg3 mice to WT levels, with statistically significant weight differences from PBS-treated Tg3 mice observed between 12–19 weeks of age for 112_S1-treated Tg3 mice (p<0.001). After 19 weeks of age, 112_S1-treated mice gained weight at a slower rate, aligning with Tg3 PBS levels thereafter. 1764_S1-treated Tg3 mice showed significant weight improvement (p<0.0001) throughout their lifespan (Figure 5B).

Tg3 mice exhibited early mortality, with a median survival probability of 32 weeks. Treatment with the E1 isoform-selective 112_S1 did not affect early mortality, resulting in a median survival probability of 31 weeks. 1764_S1-treated mice did not exhibit any signs of mortality up to 40 weeks of age, the study’s endpoint, showing survival indistinguishable from WT PBS mice (Figure 5C).

The nest-building test was conducted at 11 and 21 weeks of age. Tg3 PBS males showing lower nest manipulation scores, lower nest site scores, and higher amounts of unused nest material compared to WT PBS males at both time points. Treatment with the E1 isoform-selective 112_S1 slightly improved nest manipulation and site scores and reduced unused nest material at 11 weeks compared to Tg3 PBS mice, but these improvements were not statistically significant and were not sustained at 21 weeks of age. In contrast, Tg3 mice treated with 1764_S1 showed a complete rescue of nest manipulation and site scores and unused nest material at 11 weeks of age, maintaining these improvements at 21 weeks of age, with scores closely resembling those of WT PBS mice (Figure 5D).

In the Open Field Test, administered at 12 and 22 weeks of age, Tg3 PBS males exhibited increased distance moved, reduced vertical activity, and increased time spent in the center compared to WT PBS males. Neither 1764_S1 nor 112_S1 treatments rescued these effects. 1764_S1 treatment significantly increased vertical activity at both 12 weeks of age (p<0.001) and 22 weeks of age (p<0.0001) compared to Tg3 PBS controls, with activity levels even surpassing those of WT PBS males, indicating an overcorrection similar to that observed in Tg1 mice (Figure 5E).

The rotarod test, administered at 13 and 23 weeks of age, showed no genotype effects at 13 weeks, with similar latency to fall times for Tg3 PBS and WT PBS males. By 23 weeks of age, Tg3 PBS animals exhibited a reduced latency to fall compared to WT animals. Treatment with 112_S1 improved latency to fall in Tg3 animals at both 13 and 23 weeks, although this improvement was not statistically significant. In contrast, 1764_S1 treatment significantly increased latency to fall at 13 weeks of age (p<0.05) and 23 weeks of age (p<0.001) compared to Tg3 PBS males, with latency times even surpassing those of WT PBS males (Figure 5F). The survival probability of Tg3 mice in this study was significantly higher than in previous reports^36^. This suggests that the MDS phenotype in both Tg1 and Tg3 lines has been significantly modified since the transgenic lines were first studied. Isoform selective MECP2 modulation improved phenotypes but waned by four months without affecting survival. In contrast, non-selective treatment at six weeks yielded sustained neurobehavioral recovery and rescued premature mortality. While isoform-selective 112_S1 may mitigate over-silencing toxicity seen with 1764_S1, repeated dosing may be required to sustain benefits.

## DISCUSSION

Nucleic acid therapeutics offer curative potential for rare genetic disorders, but dosage-sensitive genes like MECP2 pose challenges, as both over- and under expression are pathogenic^2^. In MDS, excessive MECP2 silencing may induce Rett-like symptoms, necessitating therapeutics that are robust, tunable, and durable. We developed siRNAs targeting all MECP2 isoforms for severe duplication and leveraged the functional overlap of isoforms^20^ to develop E1-selective siRNAs for milder forms of MDS. A single dose achieved up to 80% mRNA knockdown at four months, with silencing durability influenced by isoform specificity and scaffold chemistry.

In Tg3 (severe model), non-selective siRNA 1764_S1 fully rescued behavior, weight loss, and survival over one year—an unprecedented outcome for MDS. E1-selective 112_S1 showed transient benefits, losing efficacy by 19 weeks, suggesting potential for use in milder cases or with repeated dosing. Tg1 (mild model) exhibited no overt phenotype, yet 1764_S1 affected behavior and weight, implying possible toxicity in absence of disease. Notably, phenotype onset in both Tg1 and Tg3 was delayed relative to published data^36^, likely due to transgenerational or environmental modifiers^39–41^. siRNA silencing durability correlated with scaffold design, consistent with literature^31,42^. A single 1764_S1 dose conferred lasting phenotypic benefit. Unlike antisense oligonucleotides (e.g., ION440)^3^ siRNA show broader brain distribution and deeper tissue penetration^43–45^, enhancing clinical appeal—especially given the risks of repeated IT or ICV delivery^8–11^ in pediatric populations.

Future development requires transcriptomic profiling to compare isoform-selective and pan-MECP2 silencing with knockout and overexpression models and defining NOAELs in multiple species. Although ~80% silencing showed no overt effects in wildtype mice, early subtle deficits cannot be excluded. Biomarkers for MECP2 engagement are also essential, with Ionis trials potentially informing future efforts. Overall, these siRNAs represent promising candidates for MDS therapy and tools for dissecting MECP2 isoform biology and silencing thresholds relevant to RTT.

## MATERIALS AND METHODS

### siRNA Selection

siRNA sequences were selected based on established rules of thermodynamics, G+C content, self-complementarity, and off-targets with seed region homology^27^. Potential siRNA sequences were further filtered based on isoform selectivity, seed region matches to off-target mRNA and lncRNA and species cross homology. Oligonucleotide synthesis, deprotection, purification for *in vitro* and *in vivo* experiments and LC-MS analysis are included in the Supplementary Materials.

### Cell culture

HeLa cells (ATCC, #CCL-2) and N2A cells (ATCC, #CCL-131) were maintained in Dulbecco′s Modified Eagle′s Medium (DMEM) (Cellgro, #10–013CV). The media were supplemented with 9% fetal bovine serum (FBS) (Gibco, #26140), and cells were grown at 37 °C and 5% CO2. Cells were split every 3 to 7 d and discarded after 15 passages.

### Animal Studies

All experimental studies involving mice were approved by the University of Massachusetts Chan Medical School Institutional Animal Care and Use Committee (IACUC) (protocol # A-2411) and the JAX In Vivo Pharmacology Services IACUC (protocol AUS #21053). Tg1 (JR#8679) and Tg3 (JR#8680) mice were obtained from the Jackson laboratory. Mice were housed at up to five mice per cage (University of Massachusetts Chan Medical School) or 3 mice per cage (JAX In Vivo Pharmacology Services) with food and water ad libitum.

Detailed procedures including surgical administration of test articles and behavioral tests are included in the Supplementary Materials.

### mRNA Quantification

mRNA quantification was performed from *in vitro* cell lysate or from tissue punches stabilized in RNAlater (Invitrogen) using the QuantiGene^™^ Singleplex branched DNA assay (Invitrogen) as previously described^46^. Briefly, cells were lysed in QuantiGene^™^ Lysis Mixture and tissues biopsy punches were homogenized in QuantiGene^™^ Homogenizing Solution containing 0.2mg/mL Proteinase K according to the manufacturer’s instructions. mRNA was detected according to the QuantiGene^™^ Singleplex branched DNA assay protocol using the following probe sets: mouse HPRT (SB-15463), mouse MECP2 (SB-11697), human HPRT (SA-10030), human MECP2 (SA-14665). Custom probe sets were designed to selectively detect mouse MECP2 E1 (DR7DPC3), mouse MECP2 E2 (DR47VRG), human MECP2 E1 (DR32Z69) and human MECP2 E2 (DR2W7MC). Luminescence was detected on a Tecan M1000 (Tecan) plate reader.

### Microscopy and immunofluorescence of tissue sections

Formalin-fixed, paraffin-embedded tissue sections were deparaffinized in xylene, rehydrated, and stained with MECP2 (D43) XP Rabbit mAb #3456 at a 1:50 dilution (Cell Signaling Technology). Donkey anti-rabbit IgG (H+L) (Alexa Fluor^®^ 594) (#A-21207, Invitrogen) was used as secondary antibody at a 1:500 dilution. The nucleus was stained with DAPI (Molecular Probes). Images of three sections per mouse, 100 μm apart, were acquired with Leica DMi8 inverted tiling microscope (Leica Microsystems) and processed using LAS X.

### Image analysis and quantification of nuclei

Image analysis of immunofluorescence tissue sections were performed using the ImageJ tools. Briefly, images were loaded onto ImageJ and split into a DAPI channel and a MECP2 channel. The threshold for the DAPI signal was adjusted using the ‘adjust → threshold’ options until the DAPI signal was accurately partitioned from background. After threshold adjustment, the particle analysis function was used by going to ‘analyze → analyze particles’ to obtain a region of interest (ROI) list. Each ROI was a nucleus identified by the ImageJ software. The ROI list was saved as ‘total nuclei’. Next, the MECP2 channel was preprocessed by background subtraction using a rolling ball radius of 50 pixels.

A custom ImageJ script was run to measure the MECP2 signal per nucleus that was previously identified from the DAPI channel and is available in supplementary methods.

## Data Availability

All data are available in the main text or the supplementary materials.
